# Serum LBP Is Associated with Insulin Resistance in Women with PCOS

**DOI:** 10.1371/journal.pone.0145337

**Published:** 2016-01-22

**Authors:** Qibo Zhu, Huang Zhou, Aipin Zhang, Rufei Gao, Shumin Yang, Changhong Zhao, Yue Wang, Jinbo Hu, Richa Goswami, Lilin Gong, Qifu Li

**Affiliations:** The First Affiliated Hospital of Chongqing Medical University, 400016, Chongqing, China; Peiking university third hospital, CHINA

## Abstract

**Introduction:**

Lipopolysaccharide-binding protein (LBP) is closely associated with many metabolic disorders. However, no study has been done to explore the relationship between LBP and polycystic ovary syndrome (PCOS). The objective of this study was to investigate whether the serum LBP level is elevated and associated with insulin resistance (IR) in PCOS.

**Participants and Design:**

In this cross-sectional study, 117 PCOS patients and 121 age-matched controls were recruited. Hyperinsulinemic-euglycemic clamp was performed with an expression of M value for insulin sensitivity. Fasting serum samples were collected to detect LBP, lipids, insulin, sex hormones and high sensitive C reactive protein (hs-CRP). Pearson’s correlation and multiple linear regression was used to analyze the associations between M value and LBP level.

**Settings:**

The study was performed in a clinical research center.

**Results:**

Compared with controls, PCOS subjects had a significantly higher LBP concentration (33.03±14.59 vs. 24.35±10.31 μg/ml, p<0.001), and lower M value (8.21±3.06 vs. 12.31±1.72 mg/min/kg, p<0.001). Both in lean and overweight/obese individuals, serum LBP level was higher in PCOS subjects than that in controls. M value was negatively correlated with body mass index (BMI), fasting serum insulin, triglycerides, low-density lipoprotein cholesterol (LDL-c), free testosterone, high sensitive C reactive protein (hs-CRP) and LBP, whereas positively correlated with high-density lipoprotein cholesterol (HDL-c) and sex hormone binding globulin (SHBG). Serum LBP level was associated with M value after adjusting for BMI, fasting serum insulin, SHBG, as well as hs-CRP.

**Conclusion:**

Serum LBP level significantly is elevated in PCOS, and is independently associated with IR in PCOS.

## Introduction

Polycystic ovary syndrome (PCOS) is characterized as oligo- or anovulation, hyperandrogenism and polycystic ovaries, afflict 5%~10% of women of reproductive age, is a cause of infertility in 25%~30% women with anovulation [1].Given that PCOS is often accompanied with metabolic disorders such as insulin resistance (IR), obesity and dyslipidemia, it is a high risk factor of type 2 diabetes mellitus (T2DM), cardiovascular diseases, and endometrial cancer [2]. The estimated prevalence of IR in PCOS is 50%~80% [3]. Compared with age, BMI-matched women with normal ovulation, PCOS patients show higher serum insulin concentration and homeostasis model assessment (HOMA)-IR[4]. Additionally, elevated levels of inflammatory factors such as high sensitive C reactive protein (hs-CRP), interleukin 6 (IL-6), tumor necrosis factor (TNF-α), and complement factor 3 (C3) were observed in PCOS, implying a possible role of chronic inflammation in PCOS [5–10].

Lipopolysaccharide (LPS) was found to stimulate the release of inflammatory factors, and subsequently lead to acute and/or chronic systematical inflammation [11,12]. Several researches show that increased circulating LPS/endotoxin level is associated with dyslipidemia, obesity, and insulin resistance (IR) [12–14]. As one of the most important ligands of LPS, lipopolysaccharide-binding protein (LBP) transfers LPS to LPS receptor resulting in an active inflammatory response [15]. Several studies suggest positive correlations between serum LBP level and metabolic disorders including IR, obesity, T2DM, and metabolic syndrome (MetS) [16–18].

Whether LBP is associated with PCOS is unknown. Thus our study aims to explore the associations between serum LBP levels and PCOS and IR in PCOS.

## Subjects and Methods

### Subjects

One hundred and seventeen women with PCOS were recruited from patients who visited the First Affiliated Hospital of Chongqing Medical University between October 2010 and August 2012.A total of 121 women of similar age with normal menstrual cycles, and no clinical and/or biochemical hyperandrogenism were set as controls. Exclusion criteria for both groups included pregnancy, history of diabetes, and the use of hormonal medications and/or antidiabetic drugs within the past 3 months. The study was approved by the First Affiliated Hospital of Chongqing Medical University Ethical Committee, and all subjects gave informed consent.

### Data Collection

A Standard questionnaire was provided to collect health information. All patients underwent anthropometric measurements: height, weight, and waist circumference (WC), blood pressure was recorded in a sitting position after a minimum 5-min rest (in the right arm). Body mass index (BMI) was determined as the ratio between weight and the square of height (kg/m^2^).

### Laboratory Procedures

Oral glucose tolerance test (OGTT) was performed between the 5^th^day to the 8^th^ day of menstrual period. Venous blood samples were collected after an overnight fast for further tests. Plasma glucose levels were measured with a hexokinase glucose-6 phosphate dehydrogenase method by biochemical analyzer (BS-380, Mindray Medical International Ltd. Shenzhen, China). Fasting serum insulin (FINS), total testosterone (TT), and sex hormone binding globulin (SHBG) were determined with an electrochemiluminescence method by immunoassay analyzer (DXI 800, Beckman Coulter Inc., Carlsbad, USA). Serum lipids including total cholesterol (TC), triglycerides (TG), high-density lipoprotein cholesterol (HDL-c), and low density lipoprotein cholesterol (LDL-c) were measured enzymatically on an automatic analyzer (AU5400, Olympus Co., Tokyo, Japan). Serum LBP levels were determined by LBP sandwich ELISA kit (CKH 113, Cell Science, Inc. Canton, MA) with the intra-assay CV of 9.8–17.8% and interassay CV of 6.1%.High sensitive C reactive protein (hs-CRP) was measured with a rate immune scatter turbidimetry method (Beckman Coulter Inc., Carlsbad, USA). The intra- and inter-assay coefficients of variation (CV) were 5.0 and 7.5%respectively.

### IR index

HOMA2-IR: HOMA2-IR was used to estimate IR in all participants. HOMA Calculator v2.2.2 was available onhttp://www.dtu.ox.ac.uk.

### Hyperinsulinemic-Euglycemic Clamps

Hyperinsulinemic-euglycemic clamps were performed 5–7 days after the OGTT were used to assess insulin sensitivity for all PCOS subjects and 18 controls [19]. Every clamp lasted for 180 minutes, and was carried out according to following modifications: after overnight fasting, IV catheters (18GA) were placed in both arms of subjects for insulin and glucose infusion and for blood sampling. The insulin infusion was initiated with a 10-minute priming dose of short-acting human insulin (Humulin, Lilly, France), and then maintained at a rate of 120 mU/m^2^/min for the remaining period. A variable infusion of 20% glucose was used to maintain the plasma glucose concentrationofaround5.2 mmol/l. Blood samples were obtained at 5-minute intervals to measure plasma glucose. Plasma glucose was calculated according to the glucose oxidase method using the Biosen 5030 Glucose Analyzer (EKF Industrie, ElektronikGmbH, Barleben, Germany). Insulin sensitivity was expressed as an M value (mg/min/kg), which was calculated from the glucose infusion rates during the 120~180 minutes of the hyperinsulinemic-euglycemic clamp.

### Diagnostic criteria

The diagnosis of PCOS was based on the 2003 Rotterdam consensus (The Rotterdam ESHRE/ASRM sponsored PCOS consensus workshop group) and required at least two of the following features [20]: (1) oligomenorrhea or chronic anovulation,(2) clinical and/or biochemical hyperandrogenism, and (3) ultrasound visualization of polycystic ovaries (PCO). Other known causes of hyperandrogenemia and ovulatory dysfunction should be excluded—congenital adrenal hyperplasia, Cushing’s syndrome, 21-hydroxylase deficiency, androgen-secreting tumors, hyperprolactinemia, and thyroid disease.

According to “The guidelines for prevention and control of overweight and obesity in Chinese adults”, BMI≥24kg/m^2^ is defined as overweight, while BMI≥28kg/m^2^ is defined as obese [21].

### Statistical Analysis

All analyses were conducted using statistical software SPSS 20.0. Log transformations were performed for data of skewed distribution. Independent sample t-test was used in comparisons between the two groups. Pearson’s correlation and multiple linear regression analyses were used to analyze the associations of M value and the other factors. P values<0.05 (two-tailed) were considered statistically significant.

## Results

**Fig 1:**
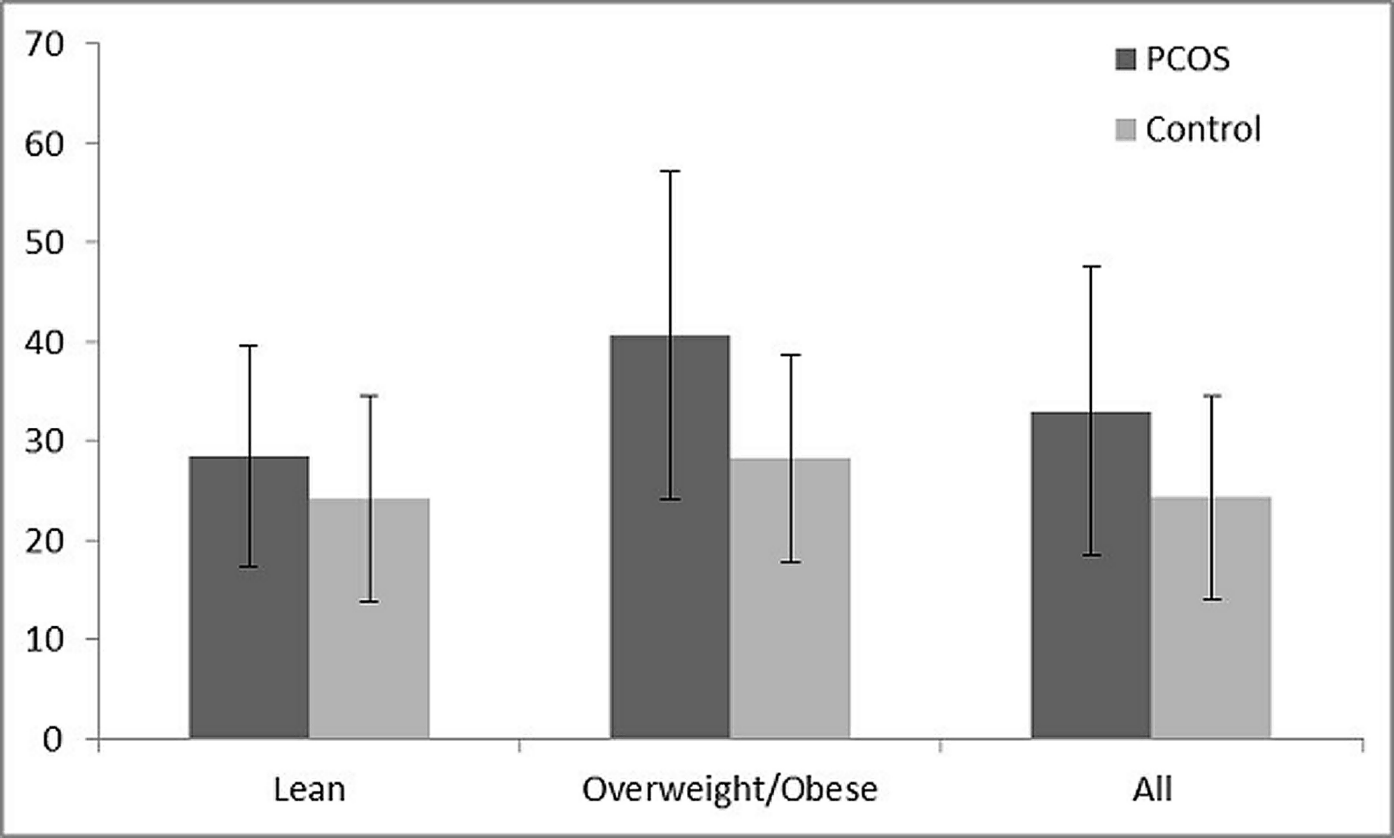
Serum LBP concentrations between PCOS and controls.

There was no significant difference in age between the two groups. Compared with the controls, PCOS subjects had a significantly higher BMI, WC, fasting plasma glucose (FPG), FINS, HOMA-IR, TG, hs-CRP, and LBP(33.03±14.59 vs. 24.35±10.31 μg/ml, p<0.001, Fig 1).A decreased M value (8.21±3.06 vs. 12.31±1.72 mg/min/kg, p<0.001), SHBG (60.19±53.31 vs. 61.66±23.59 nmol/l, p<0.001), and HDL-c were also observed in PCOS. However, no significant differences were found in TC, LDL-c, or DHEAS between the two groups(Table 1).

**Table 1:** Characteristics of subjects.

	PCOS(n = 117)	Control(n = 121)	P value
Age(years)	25.1±4.0	26.0±3.1	0.068
BMI(kg/m^2^)	23.3±4.5	20.4±1.7	<0.001
WC (cm)	78.8±11.0	70.5±5.7	<0.001
FPG(mmol/l)	5.2±0.6	4.8±0.4	<0.001
2h-PPG(mmol/l)	7.0±2.1	5.5±0.9	<0.001
FINS(mIU/l)	9.9±10.6	5.2±2.4	<0.001
HOMA2-IR	1.37±0.98	0.68±0.30	<0.001
M(mg/min/kg)^a^	8.21±3.06	12.31±1.72	<0.001
TG(mmol/l)	1.39±1.21	0.79±0.32	<0.001
TC(mmol/l)	4.21±0.92	4.19±0.72	0.810
LDL-c (mmol/l)	2.19±0.71	2.16±0.51	0.678
HDL-c (mmol/l)	1.16±0.29	1.40±0.25	<0.001
SHBG(nmol/l)	60.19±53.31	61.66±23.59	0.001
TT(ng/ml)	0.61±0.22	0.46±0.16	<0.001
FT(pg/ml)	10.16±6.24	5.92±2.80	<0.001
hs-CRP(mg/l)	1.13±1.61	0.53±0.90	<0.001
LBP(μg/ml)	33.03±14.59	24.35±10.31	<0.001

Data are presented as mean±SD and analyzed by independent sample t-test; triglycerides (TG), fasting serum insulin (FINS), fasting plasma glucose (FPG), and 2-hour postload plasma glucose (2h-PPG) werelog-transformed before comparison. FT, free testosterone was calculated by formula available from http://www.issam.ch/freetesto.htm

^a^all of PCOS women and 18 control subjects underwent hyperinsulinemic-euglycemic clamp.

Each group was divided into two subgroups (lean and overweight/obese) according to the BMI (≤24 kg/m^2^, ≥24kg/m^2^). The serum LBP level was significantly higher in overweight/obese PCOS compared to the lean subgroup (40.59±16.58 vs. 28.47±11.09 μg/ml, p<0.001)(Fig 1). In the lean group, the serum LBP level was significantly higher in PCOS than in controls (28.47±11.09 vs. 24.22±10.33 μg/ml, p = 0.005) (Fig 1), whereas the M value (9.71±2.58 vs. 12.31±1.72 mg/min/kg, p<0.001) was significantly decreased in PCOS. In the overweight/obese group, higher serum LBP levels was observed in the PCOS subjects without statistical significance (40.49±16.58 vs. 28.25±10.46μg/ml, p = 0.123) (Fig 1, Table 2).

**Table 2:** Subgroup analysis.

	Lean		Overweight/Obesity	
Variables	PCOS (n = 73)(n = 73)	Control (n = 117)(n = 117)	PCOS (n = 44)(n = 73)	Control (n = 4)(n = 117)
Age(years)	24.94±3.83	25.85±2.94	25.41±4.27	29.75±4.79
BMI(kg/m2)	20.52±1.86	20.21±1.46	28.07±3.58	25.57±1.29
M(mg/min/kg)	9.71±2.58^a^	12.31±1.72	5.73±1.99	—
LBP(μg/ml)	28.47±11.09^a^	24.22±10.33	40.59±16.58^b^	28.25±10.46

In the lean group, the serum LBP level was significantly higher in PCOS than in controls, whereas the M value was significantly decreased in PCOS. In the overweight/obese group, higher serum LBP levels were observed in the PCOS subjects without statistical significance.

a, P<0.01 compared with BMI-matched control group.

b, P<0.01 compared with lean PCOS group.

**Fig 2:**
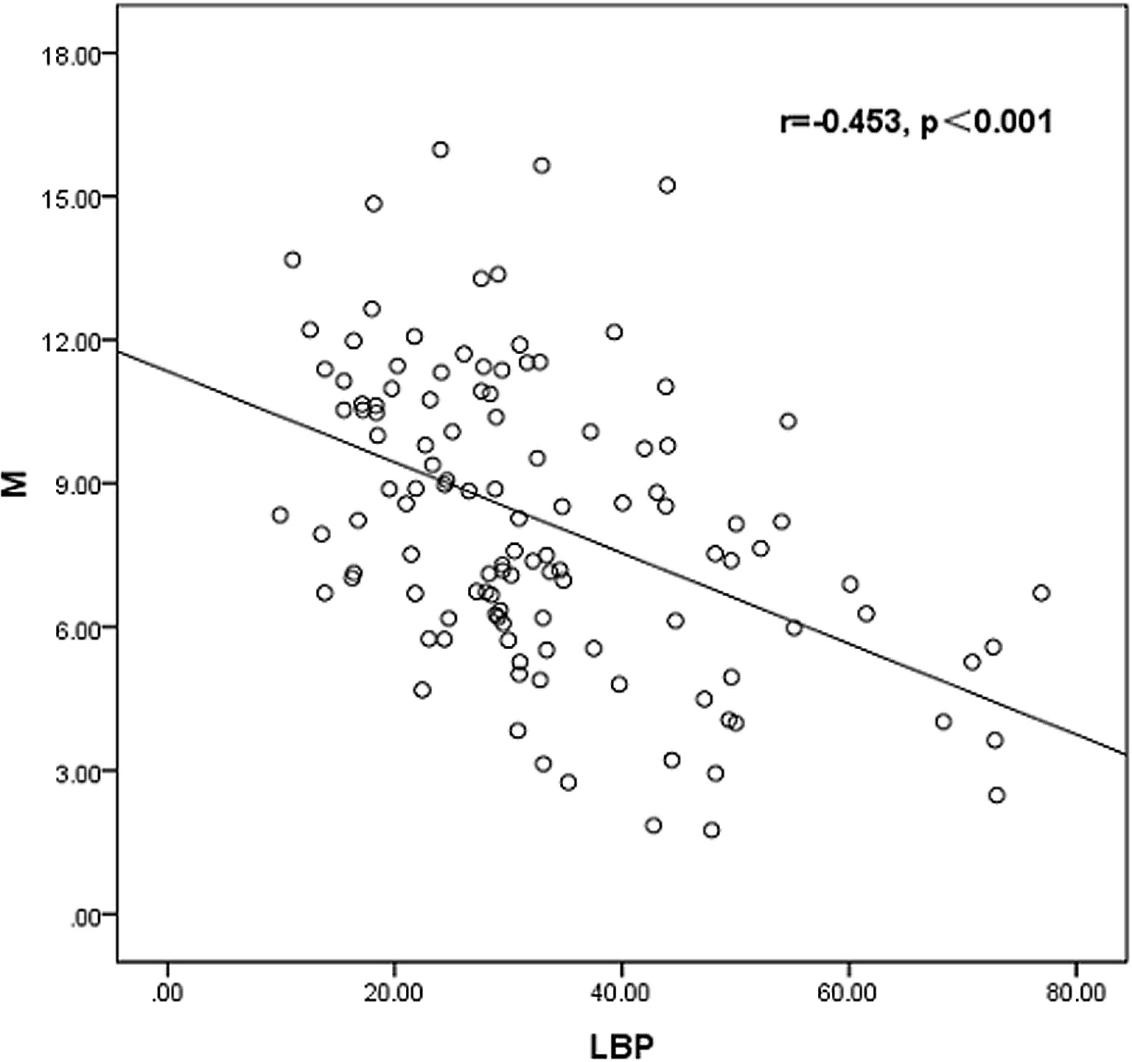
Linear correlations between LBP level and M value.

Pearson correlation analysis showed that M value was negatively correlated with BMI, WC, FINS, TG, LDL-c, FT, hs-CRP (r = -0.498, p<0.001), and LBP (r = -0.453, p<0.001), whereas Positive correlation was seen with HDL-c and SHBG (r = 0.285, p = 0.002) in PCOS patients(Fig 2).

In multiple linear regression analysis, M value was associated with the serum LBP level (standardized coefficientβ = -0.167, p = 0.039) even after adjusting for age, BMI, TG and hs-CRP in PCOS patients. In the model withHOMA2-IR as a dependent variable, after adjusting for age, BMI, TG and hs-CRP, HOMA2-IR was still associated with the serum LBP level (standardized coefficientβ = -0.271, p = 0.005) as well.

## Discussion

To our knowledge, this is the first time to investigate the association between LBP level, and IR in PCOS patients. Our results show that serum LBP level significantly increases in PCOS compared with BMI-matched controls. According to Pearson correlation analyses and multiple linear regression analyses, LBP is associated with IR in women with PCOS, independent of BMI, FINS, SHBG, as well as hs-CRP.

An epidemiological study of Spain suggests that serum LBP level is positively associated with obesity and metabolic syndrome (MetS) [10]. Another study in Caucasians shows T2DM and IR patients have higher serum LBP levels compared to healthy individuals(53.4 vs. 12.96μg/ml, p<0.001)[11]. The increasing risks of developing MetS (OR 3.54 95%CI 2.05–6.09) and T2DM (OR 5.53, 95%CI 2.64–11.59, p<0.001)were also found across the quartiles of plasma LBP levels in the Chinese population after being adjusted for factors including BMI(n = 1059) [12]. This proves that LBP plays a possible role in a series of metabolic disorders. In this study, serum LBP levels were higher in PCOS subjects than in controls in both lean and overweight/obese individuals, suggesting that the association between LBP and PCOS is independent of obesity.

Elevated circulating LBP levels in IR patients have been observed in some studies. Kheirandish-Gozal investigated the association between Obstructive Sleep Apnea (OSA) and obesity in children, and found that LBP was positively correlated with HOMA-IR (r = 0.757, p<0.001) in the whole sample [22]. Additionally, in Moreno-Navarrete et al.’s study, frequently sampled intravenous glucose tolerance test was used to assess insulin sensitivity in healthy adults and T2DM patients respectively [11]. It was found that insulin sensitivity decreased (0.58 vs. 0.22, p<0.001) in T2DM, along with an increase in serum LBP levels. In this study, we conducted the hyperinsulinemic-euglycemic clamp, a ‘gold standard’ to assess insulin sensitivity, to explore the association between LBP and IR in PCOS [23]. Given that LBP is closely related with IR in PCOS independently of BMI and FINS, it may be due to a specific type of signaling pathway that endotoxemia stimulates hyperinsulinemia. Researches done in vivo and vitro suggest that endotoxemia increases phosphorylation of Jun N-terminal kinase (JNK), expression of monocyte chemoattractant protein-1 (MCP-1) and IL-6 in muscle and adipose tissues. Endotoxemia also subsequently decreases phosphorylation of insulin receptor substrate-1 (IRS-1) and protein kinase B (Akt), finally leading to dysfunction of glucose transport [9,24].

Recently, Tremellen et al. posited the theory of “Dysbiosis of Gut Microbiota (DOGMA)” in the pathogenesis research of PCOS [25]. It was suggested that poor eating habits such as high-fat or high-fructose meals are able to increase intestinal permeability, stimulating a cascade of enterogenous lipopolysaccharide (LPS) into the blood circulation, and subsequently leading to immune/inflammatory responses. The insulin signaling pathways may be involved in inflammation leading to hyperinsulinemia, which further stimulates secretion of androgen by ovaries and disturbs follicular development [26]. Characteristics of PCOS—ovulatory dysfunction, hyperandrogenism, abnormal ovarian morphology and endotoxinemia are reasonably connected with each other in this hypothesis, suggesting that imbalance of intestinal flora may be the co-activator of inflammation and insulin signaling pathway in PCOS. We investigated the association between LBP—a ligand of LPS and PCOS, and provided evidence on this hypothesis to some extent.

Limitations of our study, first of all, there were only 4 controls with overweight/obesity in subgroup analyses, which might contribute to the lack of significant results. However, the trend was evident here. Secondly, hyperinsulinemic-euglycemic clamp was only performed for 18 individuals in control group. However, we calculated HOMI2-IRto estimate IR for all the subjects, and the result was in accordance with the former. Moreover, this was a cross-sectional study, and further longitudinal studies are needed to establish a causal relationship between LBP and IR in PCOS.

In summary, serum LBP level significantly surges in PCOS, and is associated with IR independently of BMI, FINS, and hs-CRP. Improved understanding of the causal pathways underlying these associations may not only offer evidence for researches on the pathogenesis of PCOS but could also enable new approaches for therapeutic interventions in the future.
